# Standardized Exam of the Abdomen Protocol Through Telemedicine in the Emergency Department

**DOI:** 10.5811/westjem.62235

**Published:** 2026-09-24

**Authors:** Sarah S. Abdul-Nabi, Souraya Arabi, Hind Anan, Rasha D. Sawaya, Ahmad Zaghal, Hani Tamim, Jean-Marie Al Semaani, Zahi Hamdan, Maha Makki, Moustafa Al-Hariri, Afif Jean Mufarrij

**Affiliations:** * American University of Beirut, Department of Emergency Medicine, Beirut, Lebanon; † American University of Beirut, Faculty of Medicine, Beirut, Lebanon; ‡ American University of Beirut, Clinical Research Institute, Beirut, Lebanon; § Alfaisal University, College of Medicine, Riyadh, Saudi Arabia; ¶ Qatar University, QU Health Sector, Tamayuz Simulation Center, Doha, Qatar; || Children’s Health Ireland at Temple Street, Dublin, Ireland; # University of Balamand, Faculty of Medicine, Koura, Lebanon

## Abstract

**Introduction:**

Abdominal pain is a common emergency department (ED) presentation, and virtual assessment of such complaints remains a challenge due to the limitations of remote physical examination. The standardized exam of the abdomen protocol (SEAP) was developed to guide patients in performing a self-abdominal examination during telemedicine consultations. We aimed to evaluate agreement between patient-performed abdominal self-examinations using the SEAP and physician-performed abdominal examinations.

**Methods:**

We conducted a prospective, single-center study at the American University of Beirut Medical Center ED in Lebanon from August 2023–August 2024. Adult patients who presented with abdominal pain and met the inclusion criteria were enrolled. Each patient performed a video-guided abdominal self-examination by following a brief instructional video (SEAP). The SEAP was developed by an emergency medicine faculty at our institution using pre-established abdominal examination principles and clinical practice frameworks. The protocol was translated into a structured, two-minute instructional video. Blinded abdominal examinations were then performed by an emergency medicine resident and an attending physician. The primary outcome was agreement in abdominal tenderness across seven predefined regions, assessed using the Cohen kappa coefficient. Patient experience and feasibility measures were also collected.

**Results:**

We included a total of 103 patients. Agreement between patient-performed and physician-performed examinations was slight to fair across six of the seven abdominal regions (*κ* range, .13–.41). The highest agreement was observed in the right lower quadrant, with fair agreement between patients and residents (*κ* = .47; 95% CI, 0.30–0.64) and patients and attending physicians (*κ* = .36; 95% CI, 0.18–0.53). Agreement in the remaining regions, including the upper quadrants and flanks, remained low; right upper quadrant (*κ* = .13), left upper quadrant (*κ* = .28), and flanks (*κ* range, −0.04 to .41). Most patients (97.9%; n = 94/96) rated the instructional video as clear and easy to follow.

**Conclusion:**

Patient-performed abdominal self-examination using the SEAP demonstrated limited agreement with physician examinations across most abdominal regions. Agreement in the right lower quadrant was also fair. These findings indicate that patient-performed abdominal self-examination does not reliably reproduce physician assessment. However, they provide a foundation for further studies aimed at refining and better defining the role of patient-guided abdominal examinations in telemedicine settings.

## INTRODUCTION

Telemedicine refers to the use of communication technologies to deliver healthcare services when distance is a limiting factor.^1^ Its use expanded significantly during the COVID-19 pandemic, improving access to care and continuity for a wide range of clinical conditions.^2^,^3^ Despite its advantages, telemedicine remains limited in its ability to replicate components of the physical examination that rely on direct clinician-patient contact.

Prior telemedicine research has explored clinician-guided patient self-examination, in which clinicians instruct patients to perform elements of the physical examination on themselves during video encounters.^4^ Such approaches have been described across multiple body systems, including neurologic and musculoskeletal examinations, and have demonstrated variable feasibility and diagnostic utility.^5^,^6^ However, existing studies are heterogeneous, frequently limited to small samples, and have largely focused on feasibility or clinical decision-making rather than on reproducibility or agreement between patient-performed and clinician-performed examination findings.^4^,^5^ Moreover, while previous studies focused on triage or small-sample feasibility,^4^ our study is unique in systematically measuring the reproducibility of patient-led exam findings compared to blinded physician assessments in a high-volume emergency department (ED). Furthermore, although artificial intelligence (AI) models are currently being developed for abdominal pain diagnosis, they typically do not incorporate findings from the patient’s own physical exam.^4^,^5^ This study provides a foundation for incorporating such data.

The evaluation of abdominal pain presents a particular challenge in telemedicine, as abdominal assessment traditionally relies heavily on palpation to localize tenderness and guide diagnostic reasoning.^7^ Although abdominal pain accounts for approximately 7–10% of ED visits worldwide, telemedicine studies involving abdominal complaints have primarily focused on follow-up care, triage decisions, or isolated case reports rather than systematic evaluation of patient-performed abdominal examinations.^8^–^10^ As a result, there is no standardized, reproducible protocol for guiding abdominal self-examination during telemedicine encounters, and evidence is limited regarding how closely patient-performed abdominal findings align with physician examinations.

To address this gap, we developed the standardized exam of the abdomen protocol (SEAP), a structured, video-guided approach designed to support patient-performed abdominal self-examination during telemedicine consultations. We aimed to evaluate agreement between patient-performed abdominal self-examination using SEAP and physician-performed abdominal examination in an ED setting, and to assess the feasibility of implementing such a protocol.

## METHODS

### Study Design

This is a single-center, prospective, cross-sectional study conducted at the ED of the American University of Beirut Medical Center (AUBMC). The AUBMC is a tertiary-care academic hospital and major referral center in Lebanon, receiving an average of 55,000 ED visits annually with around 5,000 presenting with abdominal pain. The ED is staffed by a mix of emergency physicians and practitioners with extensive emergency medicine (EM) experience. This study included adult patients presenting to the ED with abdominal pain between August 2023–August 2024. This study was reviewed and approved by the international review board at the American University of Beirut under protocol number SBS-2021-0202.

**Population Health Research Capsule**

What do we already know about this issue?

*Telemedicine struggles to replicate the abdominal exam, and no validated protocol exists for patient-performed abdominal self-examination*.

What was the research question?

*Does video-guided patient self-exam of the abdomen agree with blinded physician abdominal exam in the emergency department (ED)?*

What was the major finding of the study?

*Patient–resident agreement was fair only in the right left quadrant (κ = .47; 95% CI, 0.30–0.64; P < .001), and poor in other areas*.

How does this improve population health?

*This paper establishes baseline performance and limits of patient-led abdominal self-exam to guide safer telemedicine triage in overburdened EDs*.

The study was carried out over a one-year period and involved three participant groups: patients presenting with abdominal pain; EM residents; and attending physicians.

### Selection of Participants

Patients were identified prospectively at ED triage based on “abdominal pain” listed as one of the chief complaints. Trained research team members approached eligible patients prior to evaluation by a resident or attending physician. After confirming eligibility and obtaining informed consent, patients completed the self-examination protocol before any physician-performed abdominal examination took place. Eligible patients were identified at triage by trained ED nurses who had received a prior orientation about the study. A visual reminder of inclusion criteria was placed in the triage area to support screening. In parallel, in-basket notifications in the electronic health record (EHR) (Epic Systems Corporation, Verona, WI) were configured to alert the research team in real time when a patient presented with an abdominal complaint. The research team then confirmed eligibility and approached the patient to explain the study. Participation was voluntary. Written informed consent was obtained using a LimeSurvey form (LimeSurvey GmbH, Hamburg, Germany) sent directly to the patient’s phone. A survey was specifically developed for the purpose of this study (Supplementary). All participating residents and attendings were consented prior to the start of patient enrollment.

#### Inclusion Criteria

Patients were eligible if they were ≥ 18 years of age, presented with a triage complaint related to abdominal pain, and possessed a smartphone capable of running audiovisual communication applications (such as WhatsApp, Zoom, or Viber).

#### Rationale for Inclusion of Clinically Stable Patients

Only clinically stable patients were included for two reasons. First, participation required the ability to provide informed consent, watch a two-minute instructional video, and perform a guided self-examination without delaying urgent care. Second, the study was designed to evaluate a protocol intended for real-world telemedicine and remote triage settings, where patient stability is a prerequisite for self-directed examination.

#### Exclusion Criteria

Patients were excluded if they met any of the following: disorientation or agitation; known or suspected pregnancy; severe abdominal pain with a numerical rating scale (NRS) ≥ 8; body mass index (BMI) > 35; activation of the ED sepsis protocol; hemodynamic instability (heart rate >180 beats per minute, systolic blood pressure < 60 mm Hg, mean arterial pressure < 50 mm Hg, Emergency Severity Index (ESI) level 1, or deemed unstable by a physician); prior enrollment in the study; primary psychiatric complaint; cognitive or communication impairment; altered mental status; drug or alcohol intoxication; initial physical exam performed by a medical student; active or suspected COVID-19 infection; or current employment as a healthcare worker (including nurses, physicians, or students in medical or nursing programs).

Sample size was estimated using the Cochran’s formula for finite populations. Based on a retrospective review of ED visits at the AUBMC, approximately 8% of adult ED presentations involve abdominal pain. Using a 95% confidence level and a 5% margin of error, the calculated sample size was 113 patients. We initially enrolled 113 patients. Ten patients were excluded from the final analysis due to incomplete or improperly completed abdominal examination data, resulting in a final analytic sample of 103 patients. Only participants with complete paired examination data across all three examiners were included in the agreement analyses.

Each patient underwent three sequential abdominal examinations in the following order:

### Patient-performed Standardized Exam of the Abdomen Protocol

The patient received a two-minute instructional video (Supplementary) that demonstrated how to perform a guided self-examination of the abdomen. A study team member remained present to observe and record the findings but did not assist or clarify the technique to ensure that patients relied solely on the video. Patients were asked to report whether they experienced tenderness, discomfort, or no symptoms in each of seven abdominal regions: right upper quadrant; right lower quadrant; left upper quadrant; left lower quadrant; flanks; epigastric; and suprapubic areas. The findings were recorded immediately to minimize recall bias.

The two-minute video provides patients with a step-by-step guide on performing the physical exam of the abdomen. The video starts with a text slide as follows:

This video is part of a research study about self-administered standardized abdominal exam. It is not intended to be used for medical diagnosis. You will be instructed on how to perform the exam and will be asked questions about your findings. You can repeat the video as much as you need and can pause it whenever you want. This video and the following questions ask about TENDERNESS ONLY, that is pain when PRESSING on the abdomen.

The video then transitions into a picture of a woman lying on her back with her knees bent, and the narrator states, “To perform the exam, we lie on our back and bend our knees as depicted in this picture,” The video then transitions into a live demonstration of the exam, with a video of an actor lying on his back with lines drawn on his abdomen to divide it into the four main quadrants. The narrator states here “We will first start with the upper abdomen between the ribs and the belly button.” The actor in the video places his hands on both upper quadrants and then moves his hands toward the ribs and the umbilicus accordingly. The narrator then says, “We place our hands as depicted in the video,” while the actor’s hands are placed on both upper quadrants (right hand on upper right quadrant and left hand on upper left quadrant, respectively). The narrator then says, “We press hard on the right upper side noting any pain, then we move to the left upper side pressing hard, noting any pain,” while the actor in the video presses firmly with his hands on the right upper quadrant followed by the left.

The narrator then says, “We then move to the lower half of the abdomen, under the belly button,” as the actor slides his right and left hands toward the lower right and left quadrants, respectively. The narrator then says, “We press hard on the right lower side, noting any pain. We then move to the left lower side, pressing hard, noting any pain.” Concurrently, the actor presses firmly with his hands on the right lower quadrant followed by the left. The narrator then says, “We then move to the bladder area, above the bone,” as the actor points with both index fingers to the bladder area, and the narrator continues, “where we press hard, noting any pain,” as the actor presses firmly on the bladder area with both hands.

The narrator then says the following:

Finally we move to the sides, the flanks, we grab the right side and press hard with the hand and thumb, noting any pain. We then move to the left side, pressing hard with the hand and thumb noting any pain, and we would have concluded our self-administered abdominal exam.

The actor concurrently grabs both flank areas (right and left) with his right and left hands, with his four fingers on the front of his abdomen and his thumbs on the flank area of the lower back. He then presses firmly on the right flank with his hand and thumb followed by the left side. Finally, he moves his hands away from his abdomen and to the sides of his body when the exam is over.

### Resident-performed Abdominal Exam

Following the SEAP, the assigned EM resident performed a standard abdominal examination on the patient. Residents were blinded to the patient’s SEAP findings and did not have access to the self-examination results at the time of their assessment.

### Attending-performed Abdominal Exam

Finally, the attending physician conducted a routine abdominal examination as part of the standard of care. Attending physicians were blinded to both the patient-performed SEAP findings and the resident examination findings; they were not involved in the SEAP process.

While the ED at the AUBMC is staffed by experienced practitioners, the participating resident and attending followed standard clinical abdominal examination techniques to ensure the “real-world” applicability of the comparsion. Therefore, no specific study-related training was given to clinicians to maintain blinding. All examination findings were documented independently using separate standardized forms to prevent biased results.

### Method of Measurements

We collected data through chart review, structured forms, and post-encounter surveys. Patient demographics, initial vital signs, associated clinical symptoms, treatments received, final diagnosis, and ED disposition were extracted from the EHR. Abdominal tenderness assessments were recorded for each of the seven predefined abdominal regions using standardized documentation forms completed by the patient, resident, and attending. Additionally, patients completed a structured survey evaluating the clarity, usability, and acceptability of the instructional video, their comfort with performing the self-examination, and overall satisfaction with the process. We excluded from agreement analyses participants with missing or incomplete abdominal examination data in any of the seven predefined regions for any examiner. No imputation was performed. Analyses were restricted to complete cases to ensure valid region-level agreement comparisons.

### Statistical Analysis

This was primarily a comparative study. We used descriptive statistics to summarize the data, with categorical variables presented as frequencies and percentages, and continuous variables as means with standard deviations. In the bivariate analysis, the association between final discharge diagnoses and other categorical variables was analyzed using the Pearson chi-square test, while the association with continuous variables was analyzed using the Student *t*-test. We assessed agreement between the patient-performed SEAP and attending physicians’ standard physical examination using the Cohen kappa coefficient for matched-pair analysis with 95% confidence intervals calculated to quantify the precision of the concordance across the seven abdominal regions. All statistical analyses were performed using R (RStudio, PBC, Boston, MA) with appropriate libraries and IBM SPSS Statistics (Internationnal Business Machines Corp, Armonk, NY). A two-tailed *P* < .05 was considered statistically significant.

## RESULTS

Table 1 presents the baseline demographics of patients presenting to the ED with abdominal pain during the study period. The majority were female (71.8%), resided in the capital Beirut (80%), and were insured (87.4%). The mean age was 36.24 years, with nearly half identified as current smokers (46.5%). A history of gastrointestinal surgery was reported in 19.4% of patients, and 21.4% had a prior medical history related to gastroenterology. Only 6.3% had previously used telemedicine services.

Table 2 presents the characteristics of abdominal pain and associated symptoms of patients presenting to the ED during the study period. Radiating pain was reported by 27.7% of patients, and 41.8% had a history of similar episodes. Patients were categorized based on final diagnosis into medical or surgical causes. Surgical patients were more likely to report constipation (21.7% versus 6.3%, *P* = .03), while medical patients were more likely to present with diarrhea (54.4% versus 17.4%, *P* < .001) and nausea/vomiting/loss of appetite (86.1% versus 52.2%, *P* < .001). Left lower quadrant pain was more commonly observed among surgical patients (65.2% versus 46.3%, *P* = .07).

Table 3 presents the comparison of abdominal tenderness assessments across seven pain areas between patients, residents, and attending physicians. The highest agreement was observed in the right lower quadrant, with fair kappa values between patient and resident (κ = .47; 95% CI, 0.30–0.64), patient and attending (κ = 0.36; 95% CI, 0.18–0.53), and resident and attending (κ = .55; 95% CI, 0.38–0.72), all statistically significant (*P* < .001). Fair agreement was also noted in the suprapubic area between resident and attending (κ = .41; 95% CI, 0.21–0.61; *P* < .001), and in the left flank, again between patient and attending (κ = .41; 95% CI, 0.13–0.69; *P* < .001).

Table 4 presents descriptive feedback on the instructional video used for the self-examination. Among the 103 participants, most watched the video in English (66.7%), while the rest viewed it in Arabic (33.3%). The majority watched the video only once (97.9%), and only 7.3% reported needing to pause it. Regarding usability, 71.9% strongly agreed and 26.0% agreed that the video was clear and user-friendly. Similarly, 76.0% strongly agreed and 24.0% agreed that the video length was appropriate. When asked about confidence in performing the self-exam after watching the video, 58.3% strongly agreed and 33.3% agreed that they felt confident performing the exam, while only 8.3% remained neutral.

## DISCUSSION

### Interpretation

The variability in agreement across different abdominal regions suggests that anatomical location inherently influences the reliability of clinical assessments. The fair-to-moderate agreement in the right lower quadrant is encouraging for telehealth triage in the setting of appendicitis; however, the poor agreement in other areas underscores a critical gap in patient-led diagnostics. Accordingly, exploring methods to improve patient accuracy in their assessments would help avoid missing surgical diagnoses while using telehealth in the future. These findings may be driven by the fact that patients lack the clinical training to distinguish between subjective pain and objective medical signs such as guarding or rebound tenderness. Moreover, the dual role of the patient as both the examiner and subject creates a psychological and physical barrier to effective deep palpation. When compounded by factors such as body habitus or the analgesic effects of pain medication, these findings suggest that while self-examination has potential for specific triage scenarios, its utility is currently limited by the inherent difficulty of objective self-palpation, an area requiring further investigation.

### Summary of Main Findings

This study evaluated a structured self-examination protocol for abdominal pain in a real-world ED setting. Most participants were young, insured women living in Beirut. The nature of abdominal pain differed between medical and surgical cases, with constipation more commonly reported in surgical diagnoses, while nausea, vomiting, and diarrhea were more frequent among medical diagnoses. Although patients were able to complete the self-performed abdominal exam independently, agreement between patient-performed and physician-performed examinations varied significantly by anatomical region. Our study demonstrated that there was fair -to-moderate agreement between patients, residents, and physicians in the right lower quadrant, where there would be concerns of appendicitis, as well as low-to-moderate agreement in the left flank and suprapubic areas. Conversely, regions such as the upper quadrants and right flank exhibited either fair or poor agreement. Feedback indicated that patients felt comfortable performing the exam and found the video easy to follow.

### Comparison to Previous Studies

While most telehealth studies to date have focused on postdischarge follow-up or imaging-related decisions,^6^ our study stands out in its evaluation of a structured, patient-performed abdominal exam. It uniquely centers the patient as an active participant in the diagnostic process by validating agreement between their self-exam and the assessments performed by physicians in the ED. While most telemedicine tools focus on postdischarge follow-up, and some aim to support triage accuracy through systems such as the ESI^10^ or imaging decision aids, very few consider the patient’s role before the initial ED visit.^6^ Our study highlights the potential of involving patients in early symptom assessment through structured self-exams.

With the global increase in ED crowding, patients are experiencing longer lengths of stay and delayed care. Furthermore, during the COVID-19 pandemic, many patients avoided seeking emergency care altogether, despite experiencing pain symptoms at home.^3^ In parallel, AI models are increasingly being developed to support diagnostic decision-making regarding abdominal pain. However, these tools are primarily designed for use by physicians and do not assess or incorporate the patient’s own physical exam findings.

Our study findings are consistent with previous reports indicating that females presented with abdominal pain and related complaints more frequently than males.^11^ Moreover, our findings reflect a clear divergence between presentations of surgical and medical cases; surgical cases were more frequently associated with presentations of constipation, while medical cases more frequently involved presentations of nausea, vomiting, and diarrhea. These findings are also consistent with those in the literature.^12^

### Strengths

A primary strength of this study is its pioneering approach as the first to evaluate a structured, video-guided abdominal self-examination protocol within a high-volume ED setting. The methodological rigor, characterized by prospective, blinded assessments by both residents and physicians, ensures a robust comparison between patient-led and professional evaluations. Furthermore, the feasibility and positive patient feedback demonstrate that the protocol is user-friendly and practical.

### Clinical Implications

These findings have important implications for health policy, particularly in low-resource and overburdened emergency care settings. For policymakers working to expand digital health infrastructure, especially in systems strained by limited capacity or crisis conditions, patient-led assessment protocols such as SEAP offer a promising and practical approach to improve access, engagement, and emergency care delivery.

### Research Implications

Although our study did not reveal strong agreement between patient- versus physician-performed abdominal exam, the potential for this approach remains high as it offers a necessary foundation for further studies aimed at refining and better defining the role of patient-guided abdominal examinations in telemedicine settings. Future research should use larger cohorts and a more balanced use distribution to validate these findings.

## LIMITATIONS

This study has several limitations. First, although it was conducted in the largest tertiary ED in the region with a diverse patient population, it was a single-center study, which may affect generalizability. In addition, we included only stable patients, as the exam requires a minimum level of participation. This may have led to selection bias. Access to a smartphone was needed to view the video, but this reflects current norms. Moreover, since the study sample consisted mainly of young female patients, further research should be conducted on more diverse populations including comparable proportions of males and females across a wider age range.

Because the primary objective of our study was to establish baseline agreement for SEAP against the current gold standard (physical exam), no negative or positive controls were used in this study. Future studies should include a control group (eg, patients performing a self-exam without the video guide) to isolate the specific impact of the SEAP instructional tool. Additionally, the validation and generalizibilty of the findings may be limited as the physical exam was performed by only one attending and one resident. Moreover, the exclusion of patients with a BMI > 35, while necessary for initial protocol safety and clarity, limits the generalizability of our findings to patients with higher BMI. Finally, we did not compare pain location to final diagnosis since the aim was to assess whether patients could identify tenderness in agreement with physicians. This helps determine whether they need to present to the ED and supports the development of safe and effective telehealth triage.

## CONCLUSION

This prospective study is the first to evaluate a structured, video-guided self-examination protocol for abdominal pain across all quadrants in the ED. While patients were able to complete the exam independently, only fair-to-moderate agreement with physician findings was observed, and only in the right lower quadrant, an area critical for identifying potential surgical conditions like appendicitis. The video was easy to use, could be paused or replayed, and was well-received by patients, supporting its feasibility in real-world settings. This finding highlights the need for effective tools to triage abdominal pain remotely, especially in overwhelmed healthcare systems. By advancing a structured and practical approach to remote abdominal assessment, our findings contribute to expanding telemedicine capabilities and improving ED efficiency, especially during public health crises and patient surges. Nevertheless, these findings are preliminary and require further validation with a larger sample size.

## Supplementary Information



## Figures and Tables

**Table 1:** Demographic characteristics of patients presenting to the emergency department with abdominal pain in a study evaluating agreement between patient-performed and physician-performed abdominal examination.

		Total N = 103
Sex	Female	74 (71.8%)
Age, years	Mean (SD)	36.24 (16.79)
Weight in kg	Mean (SD)	70.60 (16.32)
Smoking	Yes	47 (46.5%)
Alcohol	Yes	25 (25.5%)
Marital status	Single	52 (52.5%)
	Married	47 (47.5%)
Education	Elementary/high school/technical	8 (7.8%)
	Higher education	79 (76.7%)
Place of residence	Beirut	72 (80.0%)
Guarantor	Insured	90 (87.4%)
Previous use of telemedicine	Yes	6 (6.3%)
Past surgical history	Gastroenterology	20 (19.4%)
	Gynecologic	10 (9.7%)
	Urology	5 (4.9%)
	Other*	21 (20.4%)
Past Medical history	Gastroenterology	22 (21.4%)
	Cardiovascular	14 (13.6%)
	Diabetes	7 (6.8%)
	Stroke	1 (1.0%)
	Kidney stones	9 (8.7%)
	Gynecological disease	6 (5.8%)
	Other^**^	12 (11.7%)

* Other: tonsillectomy; septoplasty; submucous resection; rhinoplasty; nasal.

**Table 2:** Description of abdominal pain nature and associated symptoms in patients presenting to the emergency department stratified by surgical and medical diagnosis.

		Total N = 103	Surgical n = 23	Medical n = 80	*P* value
Duration (hours)	Mean (SD)	24.57 (26.17)	18.65 (23.47)	26.28 (26.80)	.23
Abdominal pain radiation	Yes	28 (27.7%)	9 (39.1%)	19 (24.4%)	.16
Previous similar pain	Yes	41 (41.8%)	13 (56.5%)	28 (37.3%)	.10
Medication taken at home	Yes	53 (51.5%)	14 (60.9%)	39 (48.8%)	.30
Associated symptoms	Fever	21 (20.8%)	3 (13.6%)	18 (22.8%)	.35
	Constipation	10 (9.8%)	5 (21.7%)	5 (6.3%)	.03
	Diarrhea	47 (46.1%)	4 (17.4%)	43 (54.4%)	< .001
	Blood in stool	1 (1.0%)	0 (0.0%)	1 (1.3%)	.59
	Dysuria	6 (5.9%)	1 (4.3%)	5 (6.3%)	.72
	Back/flank pain	19 (18.6%)	6 (26.1%)	13 (16.5%)	.30
	Nausea/vomiting/loss of appetite	80 (78.4%)	12 (52.2%)	68 (86.1%)	< .001
	Bloating	31 (30.4%)	9 (39.1%)	22 (27.8%)	.30
	Other*	36 (35.3%)	9 (39.1%)	27 (34.2%)	.66
Pain area	Right upper quadrant	35 (14.6)	8 (34.8)	27 (33.8)	.93
	Left upper quadrant	33 (32.0)	5 (21.7)	28 (35.0)	.23
	Left lower quadrant	50 (48.5)	15 (65.2)	35 (43.8)	.07
	Right lower quadrant	48 (46.6)	11 (47.8)	37 (46.3)	.89
	Suprapubic	32 (31)	10 (43.5)	22 (27.5)	.14
	Right flank	17 (16.5)	5 (21.7)	12 (15.0)	.44
	Left flank	15 (14.6)	3 (13.0)	12 (15.2)	.80

* Other: chest pain; cough; dizziness; headache; rhinorrhea; shortness of breath; sore throat.

*SD*, standard deviation.

**Table 3:** Comparison of pain areas between patient versus resident/patient versus attending/resident versus attending exams.

		Patient vs Resident N = 103		Patient vs Attending N = 103		Resident vs Attending N = 103	

		No Tenderness	Tenderness	No Tenderness	Tenderness	No Tenderness	Tenderness
Pain Area # 1 RU	No Tenderness	53 (52.0%)	14 (13.7%)	52 (52.5%)	13 (13.1%)	60 (61.2%)	12 (12.2%)
	Tenderness	22 (21.6%)	13 (12.7%)	23 (23.2%)	11 (11.1%)	14 (14.3%)	12 (12.2%)
	*P value*	.08		.17		< .001	
	*Kappa (95% CI)*	0.17 (−0.03 to 0.37)		0.13 (−0.06 to 0.33)		0.30 (0.09 to 0.51)	
Pain Area # 2 LU	No Tenderness	58 (56.3%)	12 (11.7%)	61 (61.6%)	7 (7.1%)	65 (65.7%)	9 (9.1%)
	Tenderness	19 (18.4%)	14 (13.6%)	20 (20.2%)	11 (11.1%)	16 (16.2%)	9 (9.1%)
	*P value*	< .001		< .001		< .001	
	*Kappa (95% CI)*	0.27 (0.07–0.47)		0.28 (0.08–0.48)		0.26 (0.05–0.48)	
Pain Area # 3 RL	No Tenderness	43 (42.2%)	9 (8.8%)	40 (40.4%)	9 (9.1%)	49 (50.0%)	8 (8.2%)
	Tenderness	18 (17.6%)	32 (31.4%)	23 (23.2%)	27 (27.3%)	13 (13.3%)	28 (28.6%)
	*P value*	< .001		< .001		< .001	
	*Kappa (95% CI)*	0.47 (0.30–0.64)		0.36 (0.18–0.53)		0.55 (0.38–0.72)	
Pain Area # 4 LL	No Tenderness	40 (39.2%)	14 (13.7%)	43 (43.4%)	10 (10.1%)	47 (48.0%)	11 (11.2%)
	Tenderness	20 (19.6%)	28 (27.5%)	21 (21.2%)	25 (25.3%)	16 (16.3%)	24 (24.5%)
	*P value*	< .001		< .001		< .001	
	*Kappa (95% CI)*	0.33 (0.14–0.51)		0.36 (0.18–0.54)		0.42 (0.23–0.60)	
Pain Area # 5 Suprapubic	No Tenderness	54 (52.9%)	16 (15.7%)	57 (57.6%)	11 (11.1%)	60 (61.2%)	8 (8.2%)
	Tenderness	17 (16.7%)	15 (14.7%)	19 (19.2%)	12 (12.1%)	15 (15.3%)	15 (15.3%)
	*P value*	.01		.01		< .001	
	*Kappa (95% CI)*	0.24 (0.04–0.44)		0.24 (0.04–0.45)		0.41 (0.21–0.61)	
Pain Area # 6 RF	No Tenderness	84 (82.4%)	1 (1.0%)	80 (80.8%)	2 (2.0%)	93 (94.9%)	2 (2.0%)
	Tenderness	15 (14.7%)	3 (2.0%)	17 (17.2%)	0 (0.0%)	3 (3.1%)	0 (0.0%)
	*P value*	.02		.51		.80	
	*Kappa (95% CI)*	0.16 (−0.01 to 0.45)		−0.04 (−0.08 to 0.01)		−0.02 (−0.05 to −0.001)	
Pain Area # 7 LF	No Tenderness	82 (82.0%)	3 (3.0%)	83 (85.6%)	0 (0.0%)	89 (92.7%)	3 (3.1%)
	Tenderness	13 (13.0%)	2 (2.0%)	10 (10.3%)	4 (4.1%)	3 (3.1%)	1 (1.0%)
	*P value*	.11		< .001		.03	
	*Kappa (95% CI)*	0.14 (−0.10 to 0.37)		0.41 (0.13 to 0.69)		0.22 (−0.19 tto 0.62)	

* For agreement analyses, patient-reported “discomfort” was grouped with “tenderness” and analyzed as a binary outcome (tenderness present versus absent) to align with physician documentation.

*LF*, left flank; *LL*, left lower; *LU*, left upper; *RF*, right flank; *RL*, right lower; *RU*, right upper.

**Table 4:** Patients’ descriptive feedback of the standardized exam of the abdomen protocol video in a study evaluating agreement between patient-performed and physician-performed abdominal examination.

		Total N = 103
In what language did you watch the video?	English	64 (66.7%)
	Arabic	32 (33.3%)
How many times did you repeat the video?	Once	94 (97.9%)
	Twice	2 (2.1%)
Did you need to pause the video?	Yes	7 (7.3%)
The video is clear and user friendly	Neutral	2 (2.1%)
	Agree	25 (26.0%)
	Strongly agree	69 (71.9%)
The length of the video is appropriate	Agree	23 (24.0%)
	Strongly agree	73 (76.0%)
I feel comfortable self-examining after watching the video	Neutral	8 (8.3%)
	Agree	32 (33.3%)
	Strongly agree	56 (58.3%)
