# Supplementary material for: Standardized Exam of the Abdomen Protocol Through Telemedicine in the Emergency Department
Source: West J Emerg Med. 2026 Sep 24;27(5):1490–8. doi: 10.5811/westjem.62235 (PMC13660601; doi:10.5811/westjem.62235)
Supplement: Supplementary file 1 [file wjem-27-1490-s001.docx]

Patient’s survey

| **Demographics: معلومات ديمغرافية** | | |
| --- | --- | --- |
| Age:  العمر |  | |
| Sex:  الجنس | ☐ Male  ☐ Female  ☐ Other | ☐ ذكر  ☐ أنثى  ☐ آخر |
| Marital status:  الوضع العائلي | ☐ Single  ☐ Married  ☐ Divorced  ☐ Widowed | ☐ أعزب/عزباء  ☐ متزوج/ة  ☐ مطلق/ة  ☐ أرمل/ة |
| Educational Level:  المستوى التعليمي | ☐ No formal education  ☐ Elementary  ☐ Middle school  ☐ High school  ☐ Undergraduate  ☐ Technical  ☐ Postgraduate | ☐ لا تعليم رسمي  ☐ ابتدائي  ☐ متوسط  ☐ ثانوي  ☐ جامعي  ☐ مهني  ☐ دراسات عليا |
| Residency:  مكان السكن | ☐ Beirut  ☐ Mount Lebanon  ☐ Akkar  ☐ Baalbak-Hermel  ☐ Beqaa  ☐ Nabatiyeh  ☐ North Governorate  ☐ South Governorate  ☐ Keserwan-Jbeil | ☐ بيروت  ☐ جبل لبنان  ☐ عكار  ☐ بعلبك الهرمل  ☐ البقاع  ☐ النبطية  ☐ الشمال  ☐ الجنوب  ☐ كسروان جبيل |
| Guarantor:  التأمين الصحي | ☐ Insured  ☐ National Social Security Fund  ☐ Not insured | ☐ لدي تأمين  ☐ لدي ضمان اجتماعي  ☐ ليس لدي تأمين |

| **History: التاريخ الطبي** | | | | | | | | | |
| --- | --- | --- | --- | --- | --- | --- | --- | --- | --- |
| Weight (kg):  الوزن (كغ) | |  | | | | | | | |
| Height (cm):  الطول (سم) | |  | | | | | | | |
| Smoking status:  هل تدخنون | | ☐ Current  ☐ Previous  ☐ Never | | | | | ☐ مدخن حالي  ☐ مدخن سابق  ☐ لا أدخن | | |
| Alcohol consumption:  هل تستهلكون المنتجات الكحولية | | ☐ Current  ☐ Previous  ☐ Never | | | | | ☐ أشرب الكحول حاليا  ☐ كنت أشرب الكحول سابقا  ☐ لا أشرب الكحول أبدًا | | |
| Past surgical history:  التاريخ الجراحي | | ☐ None  ☐ Cholecystectomy  ☐ Appendectomy  ☐ C-section  ☐ Inguinal hernia repair  ☐ Umbilical hernia repair  ☐ Bariatric surgery  ☐ Intestinal obstruction repair or intestinal surgery  ☐ Other: ____________ | | | | | ☐ لا يوجد  ☐ استئصال المرارة  ☐ استئصال الزائدة الدودية  ☐ الجراحة القيصرية  ☐ إصلاح الفتق الإربي  ☐ إصلاح الفتق السري  ☐ جراحة لعلاج البدانة  ☐ إصلاح انسداد الأمعاء او جراحة الأمعاء  ☐ عمليات جراحية آخرى: ___________ | | |
| Past medical history:  التاريخ الطبي | | ☐ None  ☐ Diabetes  ☐ Dyslipidaemia  ☐ Hypertension  ☐ Coronary artery disease  ☐ Stroke  ☐ Mental disorders  ☐ Crohn’s disease  ☐ Ulcerative colitis  ☐ Appendicitis  ☐ GERD or reflux  ☐ Gastric ulcers  ☐ Biliary tract disorders (gallbladder disorders, gallstone, pancreatitis)  ☐ Irritable Bowel Syndrome (IBS)  ☐ Haemorrhoids or other anal disorders or rectal problems  ☐ Kidney stones or disorders  ☐ Previous intestinal bleed  ☐ Gynaecological diseases (such as ovarian torsion)  ☐ COVID-19  ☐ Other: ____________________ | | | | | ☐ لا يوجد  ☐ داء السكري  ☐ دهنيات بالدم  ☐ ارتفاع ضغط الدم  ☐ مرض القلب التاجي  ☐ الجلطة الدماغية  ☐ أمراض عقلية  ☐ مرض كرون  ☐ التهاب القولون التقرحي  ☐ التهاب الزائدة الدودية  ☐ ارتجاع المريء  ☐ قرحة المعدة  ☐ اضطرابات القناة الصفراوية (اضطرابات المرارة حصى المرارة والتهاب البنكرياس)  ☐ متلازمة القولون العصبي (IBS)  ☐ البواسير أو اضطرابات الشرج الأخرى او مشاكل المستقيم  ☐ حصوات الكلى أو اضطراباتها  ☐ نزيف الجهاز الهضمي السابق  ☐ الاضطرابات او الأمراض النسائية (مثل التفاف المبيض)  ☐ كوفيد -19  ☐ أمراض آخرى | | |
| **ED Visit Characteristics** | | | | | | | | | |
| Were you referred to the Emergency Department by another physician?  هل تم تحويلك إلى قسم الطوارئ من قبل طبيب آخر؟ | | | ☐ Yes  ☐ No | | ☐ نعم  ☐ كلا | | | | |
| Duration of abdominal pain (in minutes):  مدة الم البطن (بالدقائق) | | |  | | | | | | |
| Medication taken before presentation.  الأدوية التي تناولتها قبل القدوم الى المستشفى | | | 1.  2.  3.  4.  5.  6. | | | | | | |
| Abdominal pain radiation:  امتداد ألم البطن | | | ☐ No  ☐ Yes  If yes, where: _______________ | | | ☐ كلا  ☐ نعم  إذا نعم، ألى اين: _______________ | | | |
| Location of abdominal pain:  موضع ألم البطن | | | ☐ 1- Right upper section  ☐ 2- Left upper section  ☐ 3- Right lower section  ☐ 4- Left lower section  ☐ 5- Suprapubic (bladder area)  ☐ 6- Right Flank (side/kidney area)  ☐ 7- Left Flank (side/kidney area)  ☐ Generalized pain |  | | | | | ☐ 1- الربع العلوي الأيمن  ☐ 2 - الربع العلوي الأيسر  ☐ 3 - الربع السفلي الأيمن  ☐ 4 - الربع السفلي الأيسر  ☐ 5 - منطقة المبولة  ☐ 6 - الخاصرة اليمين  ☐ 7 - الخاصرة اليسار  ☐ ألم معمم |
| Degree of abdominal pain: درجة ألم البطن  **0 1 2 3 4 5 6 7 8 9 10** | | | | | | | | | |
| لا ألم | ألم خفيف ومزعج | | ألم مزعج وغير مريح | ألم مقلق، بائس | | ألم شديد ، رهيب | | أسوأ ألم ممكن ، لا يطاق ، مبرح | |
| Previous similar pain:  ألم مماثل سابق | | | ☐ Yes  ☐ No | | | ☐ نعم  ☐ كلا | | | |
| Abdominal pain description (one or more answers)  وصف ألم البطن (قد يتم تطبيق أكثر من احتمال) | | | ☐ Pressure  ☐ Aching  ☐ Squeezing  ☐ Cramping  ☐ Continuous  ☐ Episodic | | | ☐ ضغط  ☐ مؤلم  ☐ عصر  ☐ مغص  ☐ مستمر (دائم)  ☐ عرضي (متقطع) | | | |
| Associated signs and symptoms:  الأعراض المرافقة | | | ☐ Fever  ☐ Constipation  ☐ Diarrhoea  ☐ Blood in stool  ☐ Dysuria (burning on urination)  ☐ Vaginal bleeding  ☐Testicular pain  ☐ Back pain  ☐ Haematuria (blood in urine)  ☐ Nausea  ☐ Vomiting  ☐ Loss of appetite  ☐ Bloating  ☐ Chest pain  ☐ Cough  ☐ Wheezing  ☐ Sore throat  ☐ Shortness of breath  ☐ Dizziness  ☐ Headache  ☐ Rhinorrhea  ☐ Flank pain  ☐ Rash  ☐ Jaundice  ☐ Other: __________________ | | | ☐ حرارة  ☐ امساك  ☐ اسهال  ☐ دم في الخروج  ☐ حرقة عند البول  ☐ نزيف مهبلي  ☐ ألم الخصية  ☐ ألم في الظهر  ☐ دم في البول  ☐ غثيان  ☐ تقيؤ  ☐ فقدان الشهية  ☐ انتفاخ  ☐ ألم في الصدر  سعال☐  ☐ صفير في الصدر  ☐ إلتهاب الحلق  ☐ ضيق في التنفس  ☐ دوخة  ☐ صداع الراس  ☐ سيلان الأنف  ألم الخاصرة☐  ☐ طفح جلدي  ☐ يرقان  ☐ آخر: __________________ | | | |

| **After watching the video, please answer the following questions**  **بعد مشاهدة الفيديو، الرجاء الاجابة على الاسئلة اللاحقة** | | | | | | |  |
| --- | --- | --- | --- | --- | --- | --- | --- |
| **Exam Results: نتائج الفحص** | | | | | | |  |
|  | | | | Area # 1: Right upper section  ☐ No tenderness  ☐ Discomfort  ☐ Tenderness  Area # 2: Left upper section  ☐ No tenderness  ☐ Discomfort  ☐ Tenderness  Area # 3: Right lower section  ☐ No tenderness  ☐ Discomfort  ☐ Tenderness  Area # 4: Left lower section  ☐ No tenderness  ☐ Discomfort  ☐ Tenderness  Area # 5: Suprapubic (bladder area)  ☐ No tenderness  ☐ Discomfort  ☐ Tenderness  Area # 6: Right Flank (side/kidney area)  ☐ No tenderness  ☐ Discomfort  ☐ Tenderness  Area # 7: Left Flank (side/kidney area)  ☐ No tenderness  ☐ Discomfort  ☐ Tenderness | | الجزء رقم 1: الربع العلوي الأيمن  ☐ لا ألم  ☐ انزعاج  ☐ ألم  الجزء رقم 2: الربع العلوي الأيسر  ☐ لا ألم  ☐ انزعاج  ☐ ألم  الجزء رقم 3: الربع السفلي الأيمن  ☐ لا ألم  ☐ انزعاج  ☐ ألم  الجزء رقم 4: الربع السفلي الأيسر  ☐ لا ألم  ☐ انزعاج  ☐ ألم  الجزء رقم 5: منطقة المبولة  ☐ لا ألم  ☐ انزعاج  ☐ ألم  الجزء رقم 6: الخاصرة اليمين  ☐ لا ألم  ☐ انزعاج  ☐ ألم  الجزء رقم 7: الخاصرة اليسار  ☐ لا ألم  ☐ انزعاج  ☐ ألم |  |
| Other findings (bloating, etc..)  نتائج أخرى (انتفاخ ، إلخ ..) | |  | | | | |  |
| **Patients’ Experience with SEAP** | | | | | | | |
| Previous experience with telemedicine (excluding calling the doctor over the phone)  هل لديكم خبرة سابقة في التطبيب عن بعد  (باستثناء الاتصال بالطبيب عبر الهاتف) | ☐ Yes  ☐ No | | | | ☐ نعم  ☐ كلا | | |
| In what language did you watch the video?  بأي لغة شاهدت الفيديو؟ | ☐ English  ☐ Arabic | | | | ☐ الإنكليزية  ☐ العربية | | |
| How many times did you repeat the video?  كم مرة قمت بإعادة الفيديو؟ | ☐ Once  ☐ Twice  ☐ More than 2 times. Specify: _____ | | | | ☐ مرة  ☐ مرتان  ☐ أكثر من مرتين. حدد: _____ | | |
| Did you need to pause the video to understand a specific movement?  هل احتجت إلى إيقاف الفيديو مؤقتًا لفهم حركة معينة؟ | ☐ Yes  ☐ No | | | | ☐ نعم  ☐ كلا | | |
| \| Strongly Disagree لا أوافق بشدة \| Disagree لا أوافق \| Neutral محايد \| Agree أوافق \| Strongly Agree أوافق بشدة; The video to be clear and user friendly الفيديو واضح وسهل التطبيق \| 1 \| 2 \| 3 \| 4 \| 5; The length of the video is appropriate مدة الفيديو كانت جيدة \| 1 \| 2 \| 3 \| 4 \| 5; I feel comfortable self-examining after watching the video أشعر بالثقة عند إجراء الفحص الذاتي بعد مشاهدة الفيديو \| 1 \| 2 \| 3 \| 4 \| 5 | | | | | | | |
| What did you like about the “Standardized Exam of the Abdomen Protocol” video?  ماذا أعجبك في فيديو الفحص الذاتي للبطن؟ | | |  | | | | |
| What did not you like about the “Standardized Exam of the Abdomen Protocol” video?  ماذا لم يعجبك في فيديو الفحص الذاتي للبطن؟ | | |  | | | | |
| How can the video be improved?  كيف يمكن تحسين الفيديو؟ | | |  | | | | |
